# P-Solubilizing *Streptomyces roseocinereus* MS1B15 With Multiple Plant Growth-Promoting Traits Enhance Barley Development and Regulate Rhizosphere Microbial Population

**DOI:** 10.3389/fpls.2020.01137

**Published:** 2020-08-07

**Authors:** Fatima Ezzahra Chouyia, Ida Romano, Taoufiq Fechtali, Massimo Fagnano, Nunzio Fiorentino, Donato Visconti, Mohamed Idbella, Valeria Ventorino, Olimpia Pepe

**Affiliations:** ^1^Department of Biology, Faculty of Sciences and Techniques, Hassan II University, Casablanca, Morocco; ^2^Department of Agricultural Sciences, University of Naples Federico II, Naples, Italy

**Keywords:** phosphate solubilizing bacteria, biofertilizer, phosphate content, nitrogen content, plant growth-promoting rhizobacteria, plant growth

## Abstract

Phosphate-solubilizing bacteria (PSB) have been reported to increase phosphate (P) content and plant growth. Their application in agricultural systems is an eco-friendly alternative strategy for limiting negative environmental impact of chemical fertilizers and increasing costs. Therefore, the aim of this study was to isolate and characterize new putative PSB to use as inoculum to enhance plant growth and increase P bioavailability in soil. Sixteen bacteria were isolated from Moroccan oat rhizosphere and were screened for their putative P-solubilization by semi-quantitative agar spot method. The two strains MS1B15 and MS1B13, identified as *Streptomyces roseocinereus* and *Streptomyces natalensis*, respectively, showed the maximum phosphate solubilization index (PSI = 1.75 and PSI = 1.63). After quantitative assay to determine phosphate solubilization activity, *S. roseocinereus* MS1B15 was selected for evaluating its putative plant growth promotion activities including production of siderophores, indole-3-acetic acid (IAA) and amino-cyclopropane-1-carboxylate (ACC) deaminase, nitrogen fixation and antimicrobial activity against soil-borne plant pathogens. Under greenhouse condition, barley plants inoculated with *S. roseocinereus* MS1B15 significantly increased shoot and ear length as well as available phosphorus in ears and leaves and P and N contents in the soil. Overall results showed that the selected strain *S. roseocinereus* MS1B15 could represent a potential candidate as biofertilizer to increase plant growth as well as P uptake.

## Introduction

Phosphorus (P) is one of the major nutrients required by plants, indispensable in many physiological and biochemical processes. Phosphorus deficiency is a common phenomenon in worldwide agricultural soils; thus, most of the farmers regularly use chemical P fertilizers which get incorporated into the soil to avoid P limiting conditions in cropping systems. The applied P usually precipitates after the application by the formation of non-bioavailable complexes, whether in acid or alkaline soils (Urrutia et al., 2014). This mechanism generally causes a slow release of P, generating great challenges for remediation of these soils, with high accumulation of P not available to crops (Roy, 2017). Therefore, the use of phosphate solubilizing bacteria (PSB) as microbial inoculants in soils is an attractive, eco-compatible, and low-cost alternative strategy to exploit soil native P, limiting the application of chemical fertilizers with both environmental and economic benefits (ZaidiZaidi et al., 2009b).

Beneficial free-living bacteria, such as PSB, are usually referred to as plant growth-promoting rhizobacteria (PGPR) that can directly affect plant growth favoring the absorption of nutrients, such as nitrogen (N) and P, by plants (Ventorino et al., 2007; Reddy, 2013; Ahemad and Kibret, 2014). Moreover, synthesis of some phytohormones, as indol-3-acetic-acid (IAA), production of siderophores, and the ability to reduce endogenous levels of ethylene produced by plant through the enzyme 1-aminocyclopropane-1-carboxylate (ACC) deaminase, are other attributes related to plant growth promotion (Kloepper et al., 1980; Shahab et al., 2009; Glick, 2014). PGPR can affect crop growth also indirectly by preventing and reducing the effect of soil-borne plant pathogens through production of antimicrobial compounds and extracellular enzymes (Latha et al., 2009). Indeed, these beneficial microorganisms could also ameliorate plant growth and crop production in response to abiotic stress in hostile environments (Viscardi et al., 2016; Van Oosten et al., 2018).

Among known beneficial soil microbes, Actinobacteria is one of the dominant prokaryotic taxa living in the soil. These microorganisms belong to an extensive and diverse group of Gram-positive, aerobic, and filamentous prokaryotes. The most commonly described genus belonging to this taxon is *Streptomyces* which attracted special interest for plant growth-promoting (PGP) activities (Jog et al., 2012; Jog et al., 2014) and for its beneficial effects on several crop plants (Gopalakrishnan et al., 2014). In fact, some *Streptomyces* species have been reported as PGPR in some few crops such as tomato (El-Tarabily, 2008), wheat (Sadeghi et al., 2012), and chili (Passari et al., 2015). In addition, *Streptomyces* species are also well known as antifungal biocontrol agents due to their ability to inhibit the growth of several plant pathogenic fungi by producing hydrolytic enzymes (Dhanasekaran et al., 2008). Their biological control activity has been demonstrated against different soil-borne plant pathogens, such as *Fusarium* spp., *Phytophthora* spp., *Botrytis cinerea*, and *Verticillium dahlia* (Joo, 2005; de Vasconcellos and Cardoso, 2009; Sabaratnam and Traquair, 2015). Indeed, due to its different abilities, *Streptomyces* appears to be of great importance among the microbial flora in the rhizosphere.

Under this scenario, we hypothesized that the isolation of new efficient PSB with multiple PGP activities could be used as biofertilizer with great potential benefits on plants especially in poor and stressed soils. Indeed, new microbial isolates could represent potential candidates for the development strategies to gain new insight into a relevant topic as sustainable use of P and increase crop yield. Taking this into account this work aimed to isolate, select, and characterize new putative PSB with multiple PGP traits and antimicrobial activity. The selected strain was also used as bio-inoculant in a pot greenhouse experiment to evaluate its potential beneficial effect on the growth of a high-value vegetable crop such as barley, as well as its ability to increase P content in the crop and in the soil compared to un-inoculated pots.

## Materials and Methods

### Sample Collection and Bacterial Isolation

Rhizosphere samples were collected from two different sites in the northwest of Morocco (33°32′ 00″N, 7°35′00″W) in November 2018. In each field, five different oat plants were randomly selected for sampling, collected and stored at 4°C before analysis (Romano et al., 2020). The main physical and chemical properties of the rhizosphere samples are summarized in **Table 1**. For bacterial isolation, 10 g of the samples was shaken for 30 min in 90 ml of quarter strength Ringer’s solution (Oxoid, Milan, Italy) containing tetrasodium pyrophosphate (16% w/v) as previously described (Ventorino et al., 2014). Dilutions were performed from each sample followed by streaking in modified Pikovskaya’s (MPVK) without yeast extract (Nautiyal, 1999) and containing CaHPO_4_ as the only inorganic phosphate source. After incubation for 7 days at 30°C, colonies distinguished based on phenotypic features such as morphology and biochemical characteristics (Gram reaction and catalase activity) were picked from plates and purified by repetitive streaking on plate count agar (PCA, Oxoid). The isolates obtained were stored at 4°C as slant cultures for further analysis.

**Table 1:** Main properties of oat rhizosphere soils used in this study.

Soil	Texture	pH	Total carbon (g/kg)	Organic matter(g/kg)	Available phosphorus (mg/kg)	Total nitrogen (g/kg)	Nitrate (g/kg)	Ammonium (g/kg)
1	Sandy Clay Loam	7.40 ± 0.02	9.00 ± 2.26	15.50 ± 2.18	68.54 ± 3.09	0.98 ± 0.03	0.10 ± 0.00	2.62 ± 0.05
2	Sandy Clay Loam	7.10 ± 0.05	7.90 ± 1.28	13.10 ± 2.00	40.71 ± 3.10	0.87 ± 0.04	0.18 ± 0.00	1.95 ± 0.06

Data are means of three replicates ± SD.

### Screening of Efficient Phosphate Solubilizing Bacteria

The screening procedure comprised two-fold steps. For the first screening, sixteen isolates representative of different bacterial groups with similar morphological and biochemical characteristics were selected and tested *in vitro* for P-solubilizing activity on MPVK agar by semi-quantitative spot method. Inoculated plates were incubated at 30°C for 14 days, and the phosphate solubilization index (PSI) was calculated according to Gupta et al. (2012) using the formula reported by Qureshi et al. (2012). The second step was the quantitative estimation of solubilized P on MPVK liquid medium. During 15 days of incubation at 30°C in agitation (150 rpm), 1 ml of the culture was sampled every 72 h, centrifuged at 18,620*× g* for 5 min, and the supernatant was collected to measure the pH of the medium as well as to estimate the released soluble P by the molybdenum blue assay (Murphy and Riley, 1962). The concentration of P solubilized was quantified by spectroscopic absorbance measurements at 430 mµ according to the standard curve. Un-inoculated samples were used as the negative control. All experiments were performed in triplicate.

### Molecular Identification of Isolates by 16 rRNA Gene Sequencing

The selected bacterial strains were identified by the sequencing of the 16S rRNA gene. Total genomic DNA was extracted by boiling for 10 min and then was amplified by the polymerase chain reaction (PCR). The PCR mixture and conditions were performed according to Alfonzo et al. (2012) using the universal bacterial primers FD1(5′AGAGTTTGATCCTGGCTCAG3′) and RD1(5′-AAGGAGGTGATCCAGCC-3′). PCR products were purified using the QIAquick PCR Purification Kit (Quiagen, Milan, Italy) according to the manufacturer’s instructions and were sequenced as reported by Ventorino et al. (2017). The DNA sequences were compared to the GenBank nucleotide data library using the BLAST software at the National Centre of Biotechnology Information website (http://www.ncbi.nlm.nih.gov/Blast.cgi) to define their closest phylogenetic relatives. The 16S rRNA gene sequences obtained from bacterial strains were deposited in the GenBank nucleotide database under the accession number MT294018 and MT294019.

### *In Vitro* Plant Growth Promotion and Antimicrobial Activities

The selected bacterial strain MS1B15 was further screened for other plant growth promotion traits including production of siderophores, IAA, ACC deaminase activity, and N_2_-fixation ability. Siderophore production was carried out by quantitative assay as described by Arora and Verma (2017) using CAS reagent (Schwyn and Neilands, 1987) and was expressed as percent siderophore unit (psu) using the formula described by Payne (1993).

IAA was estimated using the Salkowski colorimetric assay as described by Gopalakrishnan et al. (2014) by spectroscopic absorbance measurements at 530 nm according to the standard curve.

The selected strain MS1B15 was screened for ACC deaminase activity based on its ability to grow on DF salt medium agar plates (Dworkin and Foster, 1958) containing 3 mM ACC (Sigma-Aldrich, Milan, Italy) as a sole nitrogen source according to Govindasamy et al. (2009). Plates containing DF minimal medium without ACC were used as the negative control; while DF minimal medium with (NH_4_)_2_SO_4_ (2.0 g/L) as a nitrogen source was used as positive control.

The selected strain was also tested for nitrogen fixation by PCR amplification of *nif*H gene, encoding the nitrogenase reductase enzyme. Synthetic oligo-nucleotide primers *nif*H-F,(5′-AAAGGYGGWATCGGYAARTCCACCAC-3′) and *nif*H-R,(5′-TTGTTSGCSGCRTACATSGCCATCAT-3′) (Rösch et al., 2002) were used to amplify the *nif*H gene as previously reported (Fiorentino et al., 2016).

The antimicrobial antagonism test was evaluated using the dual culture method as described by Hammami et al. (2013) against eight pathogenic eukaryotic strains belonging to the microbial collection of the Division of Biology and Protection of Agricultural and Forest Systems (Department of Agricultural Sciences, University of Naples Federico II): *Botrytis cinerea* B11, *Botrytis cinerea* B12, *Fusarium oxysporum* F3, *Fusarium oxysporum* F5, *Aspergillus niger* A31, *Phytophthora infestans* ph1, *Phytophthora cactorum* ph3, and *Phytophthora cryptogea* ph4. After incubation for 7 or 21 days at 28°C, the antimicrobial activity of the bacterial strain was highlighted by the presence of a halo around the colony without fungal growth.

### Assessment of Plant Growth Promotion Activities in Pot Greenhouse Experiment

#### Inoculum Preparation

A colony of the selected isolate was pre-inoculated in 10 ml of starch casein (SC) liquid medium (Pepe et al., 2013) and incubated for 5 days at 30°C. Culture was used to inoculate 250 ml of SC medium in 500 ml Erlenmeyer flask incubated at 30°C in a rotary shaker (150 rmp) for 5 days. After incubation, cells were harvested by centrifugation at 4732× *g* for 15 min. The recovered pellet was suspended in a peptone (5%) and sucrose (5%) solution at the ratio 1:5 (w:v) as described by Van Oosten et al. (2018) and freeze-dried. The final concentration of freeze-dried cells was 5.8 × 10^9^ CFU/g as assessed by viable counting on SC agar plates.

#### Greenhouse Experiment

Greenhouse assay was conducted from December 27^th^ 2018 to May 9^th^ 2019 in an unheated polyethylene greenhouse located at the University of Naples Federico II, Portici (NA), southern Italy (40°49′N, 14°15′E; 72 m a.s.l.).

The soil used in this experiment was a sandy clay soil (47% sand, 46% clay, 7% silt) collected from the northwest of Morocco (33°32′00″N, 7°35′00″W) with a pH of 7.36, electrical conductivity of 0.6 ds/m, organic matter of 2.32%, total N at 0.13 g/kg, carbonates at 1.35 g/kg, NO_3_-N at 39.39 mg/kg, NH_4_-N at 4.48 mg/kg, P at 119.91 mg/kg, and exchangeable K_2_O at 307.34 mg/kg.

The experimental unit was a pot, with a diameter of 15 cm, filled with 1.5 kg of unsterile soil that was thoroughly mixed, and dried in sunlight prior the start of the trial. Barley (*Hordeum vulgare* L.) seeds were sterilized with 25% (v/v) ammonia for 10 min and subsequently rinsed with sterile distilled water, followed by 10 min in 90% (v/v) ethanol, and finally they were washed six times with sterile distilled water. Four sterilized barley seeds per each experimental unit were sown directly at a depth of 2 cm. The experimental design was completely randomized with three replicates of the following treatments: un-inoculated seeds (BNOI); seeds inoculated with selected strain MS1B15 (BM). Inoculation with the strain MS1B15 was performed first as a seed-coating treatment using a 1 × 10^7^ CFU/ml cell suspension to uniformly cover the seed surface and two months after planting by fertigation with 3.33 g/pot of freeze-dried bacterial cells suspended in 100 ml of sterile distilled water.

#### Data Collection and Analysis of Plant Parameters

At the end of the experiment (134 days after sowing), all plants were harvested and separated into shoots and roots. Barley shoots were further separated into leaves, culms, and ears. Shoots’ and ears’ height was measured; all plant tissues were weighed to obtain the fresh weight and were dried in a forced-air oven at 80°C for 72 h for dry biomass determination. A sub-sample of the dried plants’ tissues was collected to determine the available phosphorus using the Olsen method (Morari et al., 2008), and total nitrogen was assessed after mineralization with sulfuric acid (96%) in the presence of potassium sulfate and a low concentration of copper by the Kjeldahl method (Bremner, 1965). After harvest a new soil characterization was performed to evaluate the effect of the inoculum on P and total N content.

#### Microbial Quantification and PCR-DGGE

At harvest, rhizosphere samples were collected from each treatment pot. Samples were suspended in quarter strength Ringer’s solution as reported above, and suitable dilutions were used to inoculate different solid media. Heterotrophic aerobic bacteria were enumerated on PCA plates and incubated for 2 days at 28°C. The enumeration of actinomycetes was performed using SC agar after incubation for 7 days at 30°C.

Total genomic DNA extraction from soil sample was performed using a Fast DNA SPIN Kit for Soil (MP Biomedicals, Illkirch, France) according to the supplier’s recommendations.

DGGE analysis was performed by using the oligonucleodide primes V3f (5′-CCTACGGGAGGCAGCAG-3′) and V3r (5′-ATTACCGCGGCTGCTGG-3′) (Muyzer et al., 1993) for prokaryotic analysis and the primers NL1 (5′-GCATATCAATAAGCGGAGGAAAAG-3′; Kurtzman and Robnett, 1998) and LS2 (5′-ATTCCCAAACAACTCGACTC-3′; Cocolin et al., 2000) to analyze the eukaryotic population. The PCR mixture and conditions for both amplifications were performed according to Ventorino et al. (2016). DGGE analyses were performed in a polyacrylamide gel using a Bio-Rad DCode Universal Mutation System (Bio-Rad Laboratories, Milan, Italy) as previously described by Ventorino et al. (2018).

### Statistical Analyses

The data were statistically analyzed by one-way ANOVA followed by Tukey’s HSD *post hoc* for pairwise comparison of means (at *P* < 0.05) using the SPSS 21.0 software package. DGGE bands were automatically detected by Phoretix 1 advanced version 3.01 software (Phoretix International Limited, Newcastle upon Tyne, England), then a cluster analysis was executed as described by Ventorino et al. (2013). The method described by Saitou and Nei (1987) was performed to obtain the correlation matrix of DGGE patterns. To highlight the percentage of similarity (S) of the microbial community due to treatments applied, the analysis of the resulting matrix was applied using the average linkage method in the cluster procedure of Systat 5.2.1.

## Results

### Screening of Phosphate Solubilizing Bacteria

A total of sixteen isolates were evaluated for *in vitro* P solubilizing activity using MPVK agar containing CaHPO_4_ as the sole P source. Out of 16 isolates, five strains (31.3%) were able to solubilize the P showing a clear halo around the colony with a PSI value ranging from 1.17 to 1.75. The highest PSI was exhibited by the isolates MS1B15 (PSI = 1.75) followed by MS1B13 (PSI =1.63).

On the basis of this preliminary screening, the isolates MS1B15 and MS1B13 were selected for further investigations and identified by 16S rRNA gene sequencing. Using the BLAST software, the nearly full-length gene sequence of the bacterial strains MS1B15 and MS1B13 showed 98.69% identity to *Streptomyces roseocinereus* and 99.59% identity to *Streptomyces natalensis*, respectively.

**Figure 1:**
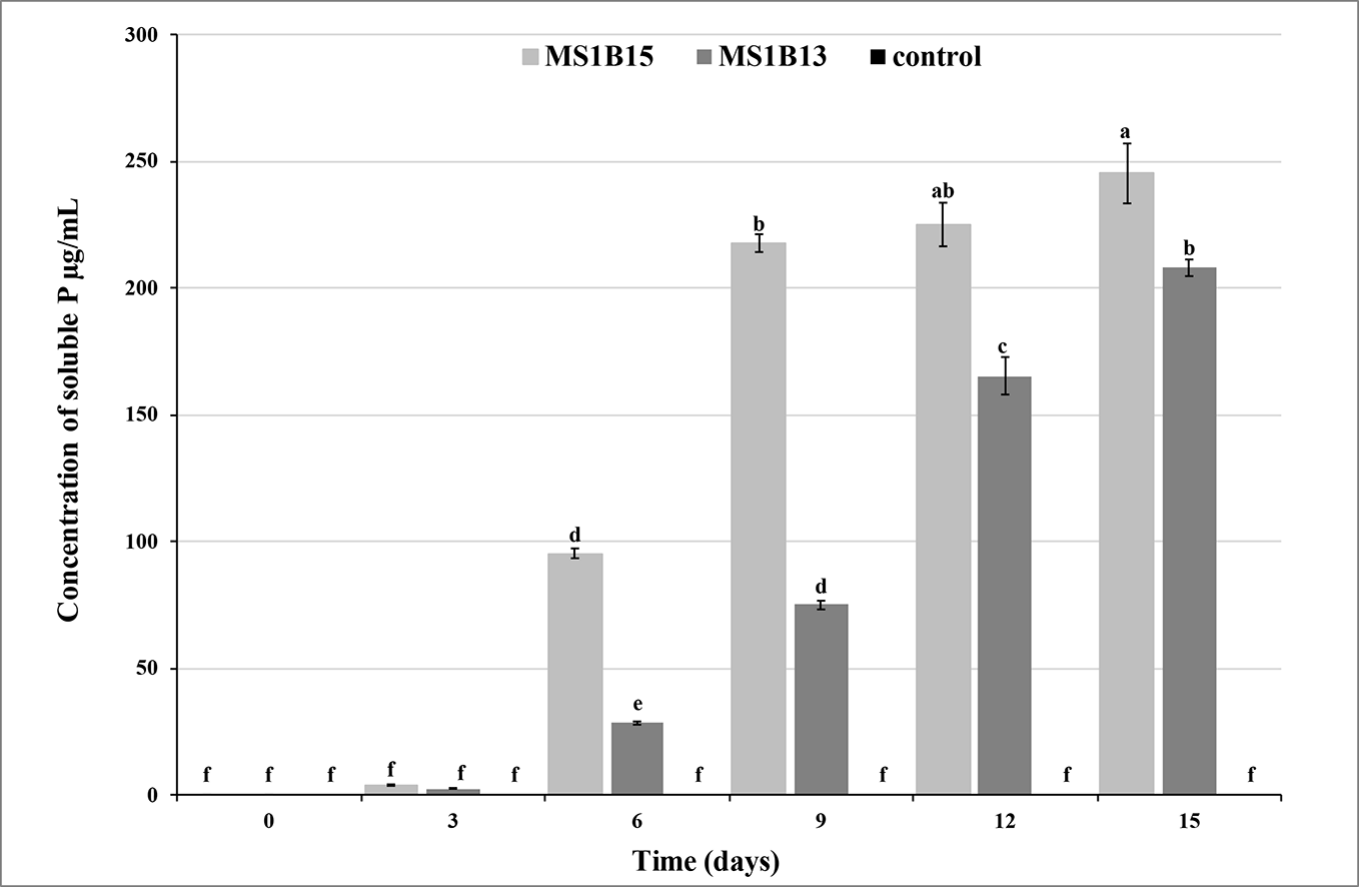
Phosphate solubilizing activity of MS1B15 and MS1B13 isolates during 15 days of incubation in MPVK liquid medium. Different letters indicate significant differences (*P* < 0.05).

Quantitative assay in liquid medium confirmed that *S. roseocinereus* MS1B15 and *S. natalensis* MS1B13 had high P-solubilizing efficiency. The soluble P concentration was slow during the first three days, after that it gradually increased reaching a value of 245.6 ± 11.8 mg/L and 207.9 ± 3.3 mg/L for MS1B15 and MS1B13, respectively (**Figure 1**). Maximum P solubilization was observed by *S. roseocinereus* MS1B15 which is consistent with the highest PSI. It has been also found that the soluble-P concentration increased as the pH decreased in liquid medium from an initial pH of 7.00 to 5.55 ± 0.11 and 6.13 ± 0.06 by MS1B15 and MS1B13, respectively. Neither soluble P (**Figure 1**) nor a decrease in pH (7.00) was detected in the control treatment.

### Characterization of Plant Growth Promotion and Antimicrobial Activities

On the basis of the results obtained by quantitative assay in liquid medium, the strain *S. roseocinereus* MS1B15 was selected for further characterization as other plant growth promotion activities and antimicrobial ability.

Quantitative analysis revealed that the strain *S. roseocinereus* MS1B15 was able to produce siderophores up to 14.09 ± 1.10 psu as well as to synthesize IAA in liquid medium with and without tryptophan (1.43 ± 0.02 and 6.34 ± 0.33 mg/L, respectively). The strain *S*. *roseocinereus* MS1B15 was also found positive to the ACC deaminase test, by growing on DF agar medium amended with ACC as the sole nitrogen source; whereas it resulted negative to *nif*H gene amplifications indicating that it was unable to fix nitrogen.

Interestingly, *S. roseocinereus* MS1B15 exerted antimicrobial activity against several tested soil-borne pathogens as *Fusarium oxysporum* F3, *Botrytis cinerea* B12, *Phytophthora cactorum* ph3, and *Phytophthora cryptogea* ph4.

### Assessment of Barley Growth Promotion and Soil Properties in Greenhouse Experiment

The ability of the *S. roseocinereus* strain MS1B15 to enhance barley growth and P availability was also evaluated in a greenhouse experiment. The results showed a significant increase in most of the variables measured in this study when the seeds were inoculated with the *S. roseocinereus* as compared to the un-inoculated plants.

Plants inoculated with *S. roseocinereus* MS1B15 showed the highest shoot length (62.13 ± 0.59 cm/plant), followed by un-inoculated control (51.75 ± 2.20 cm/plant) (**Table 2**). Similarly, a significant increase in ear length was recorded in the plants inoculated with *S. roseocinereus* MS1B15 (6.46 ± 0.20 cm/plant) as compared to the control (5.85 ± 0.13 cm/plant) (**Table 2**).

**Table 2:** Effect of inoculum on different growth parameters and phosphorus (P) content of barley plants.

Plant growth parameters	Treatment		Significance
	*S. roseocinereus* MS1B15	Control	
Shoot length (cm/plant)	62.13 ± 0.59^a^	51.75 ± 2.20^b^	**
Ear length (cm/plant)	6.46 ± 0.20^a^	5.85 ± 0.13^b^	**
Culms’ number/pot	34.00 ± 0.58^b^	37.17 ± 0.10^a^	**
Ears’ number/pot	33.00 ± 0.58^a^	25.50 ± 0.29^b^	**
Leaves’ number/pot	149.00 ± 0.58^b^	161.00 ± 1.73^a^	**
Shoot fresh weight (g/pot)	122.29 ± 4.90^a^	115.05 ± 2.16^a^	*n.s.*
Ear fresh weight (g/pot)	40.57 ± 0.47^a^	38.46 ± 1.11^a^	*n.s.*
Shoot dry weight (g/pot)	43.63 ± 1.63^a^	42.11 ± 0.41^a^	*n.s.*
Ear dry weight (g/pot)	14.71 ± 0.12^a^	13.63 ± 0.44^a^	*n.s.*
P-culm (mg/kg)	713.20 ± 19.40^a^	768.65 ± 29.24^a^	*n.s.*
P-ears (mg/kg)	1,811.25 ± 71.16^a^	1,553.97 ± 91.97^b^	*
P-leaves (mg/kg)	4,071.50 ± 29.39^a^	3,638.40 ± 86.60^b^	**

The values are means of 12 plants obtained from three experimental replicates. Within each variable different letters indicate significant differences between treatments. Significance: *P < 0.05; **P < 0.01; n.s., not significant.

Furthermore, *S. roseocinereus* MS1B15 inoculation showed a significant increase in the number of ears (33.00 ± 0.58) compared to the control (25.50 ± 0.29; **Table 2**). Whereas, a significant decrease was observed in the number of culms and leaves (34.00 ± 0.58 and 149.00 ± 0.58, respectively) with respect to the un-inoculated pots (37.17 ± 0.10 and 161.00 ± 1.73, respectively; **Table 2**).

Furthermore, no significant variations in shoot and ear fresh as well as in dry weight were recorded between the plants inoculated with *S. roseocinereus* MS1B15 and those un-inoculated (**Table 2**).

Interestingly, the P content in ears and leaves of barley plants significantly increased with the inoculation of the strain *S. roseocinereus* MS1B15 (1,811.25 ± 71.16 and 4,071.50 ± 29.39 mg/kg, respectively) as compared to the control (1,553.97 ± 91.97 and 3,638.40 ± 86.60 mg/kg, respectively; **Table 2**).

Regarding the results on soil properties, at the end of the experiment a significant increase of total N in soil was recorded in the *S. roseocinereus* MS1B15 treated pots (0.17 ± 0.01 g/kg) with respect to the control (0.15 ± 0.00 g/kg; **Table 3**). The highest available P was also recovered in *S. roseocinereus* MS1B15 inoculated pots, although it was not significantly different from the control (**Table 3**).

**Table 3:** Effect of inoculum on soil properties at the end of the experiment.

Soil property	Treatment		Significance
	*S. roseocinereus* MS1B15	Control	
pH	6.76 ± 0.00	6.71 ± 0.00	*n.s.*
CE (ms/cm)	0.59 ± 0.00	0.71 ± 0.00	*n.s.*
NO_3_-N (g/kg)	11.14 ± 0.86	7.84 ± 1.04	*n.s.*
NH_4_-N (g/kg)	35.94 ± 1.72	35.56 ± 2.40	*n.s.*
Organic matter (g/kg)	2.99 ± 0.06	2.70 ± 0.13	*n.s.*
Total carbon (g/kg)	1.74 ± 0.04	1.56 ± 0.08	*n.s.*
Total N (g/kg)	0.17 ± 0.00^a^	0.15 ± 0.00^b^	*
Available P (mg/kg)	125.09 ± 2.82^a^	110.28 ± 4.20^a^	*n.s.*

Data are means of three replicates ± SD. Different letters indicate significant differences between treatments. Significance: *P < 0.05; n.s., not significant.

#### Microbial Quantification

Inoculation with *S. roseocinereus* MS1B15 significantly affected the density of the prokaryotic populations (*P*  <  0.05). The total actinomycete concentration in the rhizosphere was influenced by the *S. roseocinereus* inoculation, in which the highest significantly actinobacterial concentration was detected (6.03 ± 0.01 log CFU/g of soil; *P* < 0.05); while the un-inoculated control showed a concentration of approximately 0.5 order of magnitude lower (5.52 ± 0.04 log CFU/g of soil). Similarly, *S. roseocinereus* MS1B15 treatment led to a significant increase of aerobic heterotrophic bacteria up to 7.29 ± 0.01 log CFU/g of soil (*P* < 0.05) with respect to the un-inoculated control (6.76 ± 0.06 log CFU/g of soil, respectively).

#### Molecular Characterization of Soil Microbes

**Figure 2:**
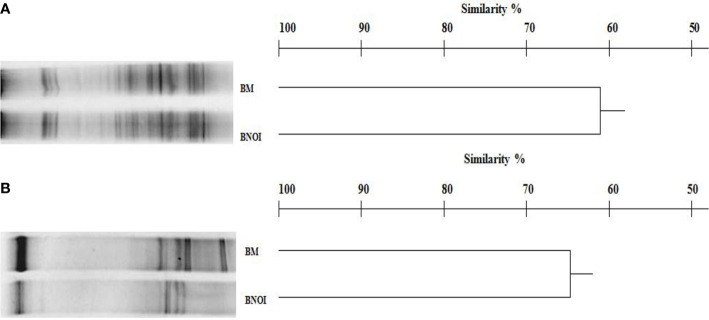
DGGE profiles and dendrogram showing the degree of similarity (%) of the prokaryotes **(A)** and eukaryotes **(B)** in the rhizosphere of barley plants. BM, pots inoculated with *Streptomyces roseocinereus* MS1B15; BNOI, un-inoculated pots.

The DGGE profile of prokaryotes in barley rhizosphere samples was complex, producing 23–28 bands as shown in **Figure 2A**. Inoculation with the PSB strain *S. roseocinereus* MS1B15 contained the highest bacterial biodiversity (28 bands). Statistical analysis on the position and intensity of the bands allowed the classification of the cluster associated to the inoculum applied to the soil and to the un-inoculated control with a similarity level equal to 62% (**Figure 2A**).

Eukaryotic populations showed simpler profiles generating 13–18 bands (**Figure 2B**). Similar to the prokaryotic results, statistical analysis of the fungal DGGE profiles determined that *S. roseocinereus* MS1B15 treatment and control, grouped with a similarity level of 65% (**Figure 2B**).

## Discussion

Recent agricultural practices tend to use sustainable technologies, including the use of microbial bioinoculants as alternative and/or with complementary functions than the use of chemical fertilizers (Fiorentino et al., 2018; Visconti et al., 2020). PSB uses are various, such as sustainable management of agricultural systems (Zaidi et al., 2009a) and promoting soil fertility (Vahed et al., 2012), enriching soil P pool and consequently providing it to the plants. These microbes could also have other interesting plant growth-promoting activities, such as the synthesis of important substances including production of phytohormones (indole-3-acetic acid) and siderophores, enhancing nitrogen fixation, and exerting antagonism against plant soil-borne pathogens (Glick, 1995).

The ecological approach developed in this study enabled the isolation of new PSB with multiple PGP activities. In particular, out of 16 isolates from the Moroccan oat rhizosphere, two strains belonging to the genus *Streptomyces* showed the best P-solubilizing activity on the solid assay as well as in the liquid assay. P-solubilizing microorganisms are active in the conversion of insoluble P to soluble forms making it accessible to plants (Rajput et al., 2013). In this study, the maximum concentration of solubilized P ranged from 207.9 ± 3.3 mg/L to 245.6 ± 11.8 mg/L, and therefore the presence of the *Streptomyce*s strains MS1B15 and MS1B13 in the rhizosphere could improve plant P assimilation. Although these values are lower than those reported by Jog et al. (2014) for *Streptomyces* sp. isolated from wheat plants (950–1,916 mg/L), they exceed the level of the other bacterial genera, including *Bacillus* and *Pseudomonas* which solubilized 128.10 mg/L and 166.53 mg/L, respectively (Habil-Addas et al., 2017; Tiwari et al., 2018).

During the growth of the two PSB strains *S. roseocinereus* MS1B15 and *S. natalensis* MS1B13, the pH of the medium decreased as soluble P increased. This result is consistent with Jog et al. (2014) who reported a strong pH decrease and soluble P increase during the growth of the two strains *S. cellulosae* and *S. tricolor*. Wei et al. (2018) observed that PSB inoculation affected pH, total acidity, and the production of several acids during composting of organic wastes suggesting that the lower pH in the PSB enriched compost than that in un-inoculated compost might be attributed to organic acids produced by microbial inoculum accompanied with the degradation of organic matter. Furthermore, Marra et al. (2011) established that there was a significantly negative linear correlation (*P* < 0.05) between culture pH and solubilized inorganic P. By observing the negative correlation between pH and soluble P (*r* = −0.940; *P* < 0.05), it could be inferred that the acidification of the medium could facilitate phosphate solubilization.

The selected strain *S. roseocinereus* MS1B15 exhibited other interesting PGP activities such as siderophore and IAA as well as ACC deaminase and antimicrobial activities. Siderophore compounds are potential plant growth promoters and disease suppressors. Khamna et al. (2009) suggested that *Streptomyces* sp. have the ability to produce hydroxymate-type siderophores, which inhibit the growth of phytopathogens by limiting iron in the rhizosphere. The strain *S. roseocinereus* MS1B15 exerted also antimicrobial activity against plant pathogenic fungi such as *Fusarium, Botrytis*, and *Phytophthora*. These results concur with other studies which have shown that several *Streptomyces* strains play a key role in protecting plants against several soil borne plant pathogens reporting them as biocontrol agents (Joo, 2005; Errakhi et al., 2007).

Previous research has documented that the *Streptomyces* genus is also able to synthesize IAA which is responsible for improving plant growth by helping it to uptake a large volume of nutrients, absorb water, increase seed germination and root elongation (El-Tarabily, 2008). According to Abd-Alla et al. (2013) who reported the ability of *Streptomyces* sp. isolated from wheat, corn, and faba bean to produce IAA in the range from 3.55 µg/ml to 22.56 µg/ml, the strain *S. roseocinereus* MS1B15 selected in this work exhibited a IAA production of 6.34 ± 0.33 µg/ml. As other bacterial genera living in the soil including *Pseudomonas* (Hall et al., 1996), *Enterobacter* (Li et al., 2000), and *Bacillus* (Ghosh et al., 2003) were able to produce ACC, also the strain *S. roseocinereus* MS1B15 was able to grow on DF agar medium amended with ACC as the sole nitrogen source. This is an interesting ability because ACC deaminase-producing bacteria have been known to promote plant growth by decreasing ethylene inhibition of various plant processes (Husen et al., 2011).

Although some *Streptomyces* isolates are able to fix atmospheric nitrogen (Sellstedt and Richau, 2013) having nitrogen-fixing genes (Dahal et al., 2017), the strain *S. roseocinereus* MS1B15 did not show positive amplification of *nif*H gene. This could be attributed to a difference in the gene sequences which did not allow the primers annealing (Gaby and Buckley, 2012).

Inoculation with bacterial strains which possess multiple PGP traits could stimulate plant growth as assessed in our experiment by the effect of *S. roseocinereus* MS1B15 on barley under greenhouse conditions. To ensure the success of the inoculum, a two-stage approach was applied in which the bacterial strain was firstly inoculated by a seed-coating treatment followed by fertigation after two months. In this way, the strain *S. roseocinereus* MS1B15 will be able to move in the plant rhizosphere due to a phenomenon known as the “rhizosphere effect” in which root-derived exudates directly affect easily degradable substances leading to proliferation of microorganisms in the rhizosphere (Ventorino et al., 2012; Gupta et al., 2016). Barley inoculated with *S. roseocinereus* MS1B15 showed an increased growth with higher shoot and ear length as well as ear number with respect to un-inoculated control. The plant growth promotion in response to inoculation with *Streptomyces* sp. has been frequently reported in various crops, such as tomato, wheat and chili (El-Tarabily, 2008; Sadeghi et al., 2012; Passari et al., 2015). Interestingly, plants treated with *S. roseocinereus* MS1B15 showed a P content in shoot tissues (ears and leaves) higher than that revealed in the control plants, highlighting the high potential of the new PSB strain *S. roseocinereus* MS1B15 to mobilize P in treated crops.

Furthermore, increasing the availability of soil nutrients is an urgent priority to meet the increasing global demand for food. In this study, the P availability and N content in the soil were increased by inoculation of *S. roseocinereus* MS1B15. Although, *S. roseocinereus* MS1B15 seems to be unable to fix nitrogen, the increase of N content in the rhizosphere inoculated with this strain could be due to the positive effect exerted on soil microflora and to the enhanced root activity of barley plant. The increase of prokaryotic populations recorded in the inoculated rhizosphere and assessed by culture-dependent and culture-independent approaches could involve also native beneficial microorganisms such as nitrogen-fixing bacteria (Woo and Pepe, 2018) and phosphate-solubilizing microorganisms. This effect could be explained by the increase of soil nutrients in treated soils. Trabelsi et al. (2012) showed that rhizobia inoculation may increase microbial diversity and structure, potentially stimulating PGPR and enhancing disease control in the soil. Similarly, in this study, the cluster analysis indicated an increase of microbial biodiversity in treated soils resulting in a low similarity with control highlighting that the rhizosphere microbial communities were affected by the inoculum applied. Moreover, root secretion induced by the PGPR may stimulate the proliferation of other bacteria in the rhizosphere soils (de-Bashan et al., 2010).

## Conclusion

The ecological approach used in this study allowed us to isolate and select the new phosphate-solubilizing strain *S. roseocinereus* MS1B15. To the best of our knowledge this is the first study reporting the ability of the *S. roseocinereus* species to solubilize phosphate. Moreover, the new selected strain *S. roseocinereus* MS1B15 showed multiple plant growth promoting activities and antimicrobial activity against several soilborne plant pathogens as well as being able to improve plant growth. The overall results highlighted that the new PSB *S. roseocinereus* MS1B15 represents a potential candidate as a biofertilizer to increase available nutrients and assimilation efficiency in sustainable agricultural crop systems. Therefore, crop inoculation with beneficial bacteria such as *S. roseocinereus* MS1B15 could be a suitable option for low-input systems, where environmental constraints and limited chemical fertilization may affect the potential yield.

## Data Availability Statement

The datasets presented in this study can be found in online repositories. The names of the repository/repositories and accession number(s) can be found below: https://www.ncbi.nlm.nih.gov/genbank/, MT294018 and MT294019.

## Author Contributions

FC performed microbiological and agronomic experiments, analyzed the data, and drafted the manuscript. IR help in the culture-dependent and culture-independent microbiological assays. TF contributed to conceiving the study. MF coordinated agronomic analyses and contributed to conceiving the study. NF and DV carried out greenhouse experiment and analyzed agronomic data. MI collected the soil samples. VV defined the experimental protocol, contributed to conceiving the study and participated in its coordination. OP coordinated microbiological analyses and contributed to conceiving the study. All authors contributed to the article and approved the submitted version.

## Funding

This work was supported by Agriges S.r.l. within the research project “BENEVEGEFIT”, MISE—Agrifood PON I&C 2014-2020.

## Conflict of Interest

The authors declare that the research was conducted in the absence of any commercial or financial relationships that could be construed as a potential conflict of interest.

## References

[B1] Abd-Alla M. H. El-Sayed E. S. A. Rasmey A. H. M. (2013). Indole-3-acetic acid (IAA) production by *Streptomyces atrovirens*. J. Biol. Earth Sci. 3, 82–93.

[B2] Ahemad M. Kibret M. (2014). Mechanisms and applications of plant growth promoting rhizobacteria: current perspective. JKSUS 26, 1–20. 10.1016/j.jksus.2013.05.001

[B3] Alfonzo A. Lo Piccolo S. Conigliaro G. Ventorino V. Burruano S. Moschetti G. (2012). Antifungal peptides produced by *Bacillus amyloliquefaciens* AG1 active against grapevine fungal pathogens. Ann. Microbiol. 62, 1593–1599. 10.1007/s13213-011-0415-2

[B4] Arora N. K. Verma M. (2017). Modified microplate method for rapid and efficient estimation of siderophore produced by bacteria. 3 Biotech. 7, 381. 10.1007/s13205-017-1008-y PMC5658296 29109926

[B5] Bremner J. M. (1965). “Total nitrogen,” in Methods of Soil Analysis. Eds. Black C. A. Evans D. D. White I. L. Ensminger L. E. (Madison, WI: American Society of Agronomy), 1149–1178.

[B6] Cocolin L. Bisson L. F. Mills D. A. (2000). Direct profiling of the yeast dynamics in wine fermentations. FEMS Microbiol. Lett. 189, 81–87. 10.1111/j.1574-6968.2000.tb09210.x 10913870

[B7] Dahal B. NandaKafle G. Perkins L. Brözel V. S. (2017). Diversity of free-living nitrogen fixing *Streptomyces* in soils of the badlands of South Dakota. Microbiol. Res. 195, 31–39. 10.1016/j.micres.2016.11.004 28024524

[B8] de Vasconcellos R. L. F. Cardoso E. J. B. N. (2009). Rhizospheric streptomycetes as potential biocontrol agents of *Fusarium* and *Armillaria* pine rot and as PGPR for *Pinus taeda*. BioControl 54, 807–816. 10.1007/s10526-009-9226-9

[B9] de-Bashan L. E. Hernandez J. P. Bashan Y. Maier R. M. (2010). *Bacillus pumilus* ES4: candidate plant growth-promoting bacterium to enhance establishment of plants in mine tailings. Environ. Exp. Bot. 69, 343–352. 10.1016/j.envexpbot.2010.04.014 25009362 PMC4084739

[B10] Dhanasekaran D. Thajuddin N. Panneerseilvam A. (2008). An antifungal compound: 4′ phenyl-1- napthyl-phenyl acetamide from *Streptomyces* sp. DPTTB16. Med. Biol. 15, 7–12. 10.1016/j.compbiomed.2012.01.007 22381026

[B11] Dworkin M. Foster J. W. (1958). Experiments with some microorganisms which utilize ethane and hydrogen. J. Bacteriol. 75, 592–603. 10.1128/JB.75.5.592-603.1958 13538930 PMC290115

[B12] El-Tarabily K. A. (2008). Promotion of tomato (*Lycopersicon esculentum* Mill.) plant growth by rhizosphere competent 1-aminocyclopropane-1-carboxylic acid deaminase-producing streptomycete actinomycetes. Plant Soil 308, 161–174. 10.1007/s11104-008-9616-2

[B13] Errakhi R. Bouteau F. Lebrihi A. Barakate M. (2007). Evidences of biological control capacities of *Streptomyces* spp. against *Sclerotium rolfsii* responsible for damping-off disease in sugar beet (*Beta vulgaris* L.). World J. Microb. Biot. 23, 1503–1509. 10.1007/s11274-007-9394-7

[B14] Fiorentino N. Ventorino V. Bertora C. Pepe O. Moschetti G. Grignani C. (2016). Changes in soil mineral N content and abundances of bacterial communities involved in N reactions under laboratory conditions as predictors of soil N availability to maize under field conditions. Biol. Fertil. Soils 52, 523–537. 10.1007/s00374-016-1095-7

[B15] Fiorentino N. Ventorino V. Woo S. L. Pepe O. De Rosa A. Gioia L. (2018). *Trichoderma*-based biostimulants modulate rhizosphere microbial populations and improve N uptake efficiency, yield, and nutritional quality of leafy vegetables. Front. Plant Sci. 9, 743. 10.3389/fpls.2018.00743 29922317 PMC5996573

[B16] Gaby J. C. Buckley D. H. (2012). A comprehensive evaluation of PCR primers to amplify the *nif*H gene of nitrogenase. PLoS One 7, e42149. 10.1371/journal.pone.0042149 22848735 PMC3405036

[B17] Ghosh S. Penterman J. N. Little R. D. Chavez R. Glick B. R. (2003). Three newly isolated plant growth-promoting bacilli facilitate the seedling growth of canola, *Brassica campestris*. Plant Physiol. Biochem. 41, 277–281. 10.1016/S0981-9428(03)00019-6

[B18] Glick B. R. (1995). The enhancement of plant growth by free-living bacteria. Can. J. Microbiol. 41, 109–117. 10.1139/m95-015

[B19] Glick B. R. (2014). Bacteria with ACC deaminase can promote plant growth and help to feed the world. Microbiol. Res. 169, 30–39. 10.1016/j.micres.2013.09.009 24095256

[B20] Gopalakrishnan S. Vadlamudi S. Bandikinda P. Sathya A. Vijayabharathi R. Rupela O. (2014). Evaluation of *Streptomyces* strains isolated from herbal vermicompost for their plant growth-promotion traits in rice. Microbiol. Res. 169, 40–48. 10.1016/j.micres.2013.09.008 24113511

[B21] Govindasamy V. Senthilkumar M. Mageshwaran V. Annapurna K. (2009). Detection and characterization of ACC deaminase in plant growth promoting rhizobacteria. J. Plant Biochem. Biot. 18, 71–76. 10.1007/BF03263298

[B22] Gupta M. Kiran S. Gulati A. Singh B. Tewari R. (2012). Isolation and identification of phosphate solubilizing bacteria able to enhance the growth and aloin-A biosynthesis of *Aloe barbadensis* Miller. Microbiol. Res. 167, 358–363. 10.1016/j.micres.2012.02.004 22417676

[B23] Gupta R. Bisaria V. S. Sharma S. (2016). Response of rhizospheric bacterial communities of *Cajanus cajan to* application of bioinoculants and chemical fertilizers: a comparative study. Eur. J. Soil Biol. 75, 107–114. 10.1016/j.ejsobi.2016.02.008

[B24] Habil-Addas F. E. Aarab S. Rfaki A. Laglaoui A. Bakkali M. Arakrak A. (2017). Screening of phosphate solubilizing bacterial isolates for improving growth of wheat. Eur. J. Biotechnol. Biosci. 6, 7–11.

[B25] Hall J. A. Peirson D. Ghosh S. Glick B. (1996). Root elongation in various agronomic crops by the plant growth promoting rhizobacterium *Pseudomonas putida* GR12–2. Isr. J. Plant Sci. 44, 37–42. 10.1080/07929978.1996.10676631

[B26] Hammami I. Hsouna A. B. Hamdi N. Gdoura R. Triki M. A. (2013). Isolation and characterization of rhizosphere bacteria for the biocontrol of the damping-off disease of tomatoes in Tunisia. C. R. Biol. 336, 557–564. 10.1016/j.crvi.2013.10.006 24296079

[B27] Husen E. Wahyudi A. T. Suwanto A. Giyanto G. (2011). Growth enhancement and disease reduction of soybean by 1-aminocyclopropane-1-carboxylate deaminase-producing *Pseudomonas*. Am. J. Appl. Sci. 8, 1073–1080. 10.3844/ajassp.2011.1073.1080

[B28] Jog R. Nareshkumar G. Rajkumar S. (2012). Plant growth promoting potential and soil enzyme production of the most abundant *Streptomyces* spp. from wheat rhizosphere. J. Appl. Microbiol. 113, 1154–1164. 10.1111/j.1365-2672.2012.05417.x 22849825

[B29] Jog R. Pandya M. Nareshkumar G. Rajkumar S. (2014). Mechanism of phosphate solubilization and antifungal activity of *Streptomyces* spp. isolated from wheat roots and rhizosphere and their application in improving plant growth. Microbiology 160, 778–788. 10.1099/mic.0.074146-0 24430493

[B30] Joo G.-J. (2005). Production of an anti-fungal substance for biological control of *Phytophthora capsici* causing phytophthora blight in red-peppers by *Streptomyces halstedii*. Biotechnol. Lett. 27, 201–205. 10.1007/s10529-004-7879-0 15717130

[B31] Khamna S. Yokota A. Lumyong S. (2009). Actinomycetes isolated from medicinal plant rhizospheric soils: diversity and screening of antifungal compounds, indole-3-acetic acid and siderophore production. World J. Microbiol. Biotechnol. 25, 649–655. 10.1007/s11274-008-9933-x

[B32] Kloepper J. W. Leong J. Teintze M. Schroth M. (1980). Enhancing plant growth by siderophores produces by plant-growth-promoting rhizobacteria. Nature 286, 885–886. 10.1038/286885a0

[B33] Kurtzman C. P. Robnett C. J. (1998). Identification and phylogeny of ascomycetous yeasts from analysis of nuclear large subunit (26S) ribosomal DNA partial sequences. Antonie Van Leeuwenhoek 73, 331–371. 10.1023/A:1001761008817 9850420

[B34] Latha P. Anand T. Ragupathi N. Prakasam V. Samiyappan R. (2009). Antimicrobial activity of plant extracts and induction of systemic resistance in tomato plants by mixtures of PGPR strains and Zimmu leaf extract against *Alternaria solani*. Biol. Control 50, 85–93. 10.1016/j.biocontrol.2009.03.002

[B35] Li J. Ovakim D. H. Charles T. C. Glick B. R. (2000). An ACC deaminase minus mutant of *Enterobacter cloacae* UW4 no longer promotes root elongation. Curr. Microbiol. 41, 101–105. 10.1007/s002840010101 10856374

[B36] Marra L. M. de Oliveira, S. M. Soares C. R. F. S. Moreira F. M. S. (2011). Solubilisation of inorganic phosphates by inoculant strains from tropical legumes. Sci. Agric. 68, 603–609. 10.1590/S0103-90162011000500015

[B37] Morari F. Lugato E. Giardini L. (2008). Olsen phosphorus, exchangeable cations and salinity in two long-term experiments of north-eastern Italy and assessment of soil quality evolution. Agric. Ecosyst. Environ. 124, 85–96. 10.1016/j.agee.2007.08.001

[B38] Murphy J. Riley J. P. (1962). A modified single solution method for the determination of phosphate in natural waters. Anal. Chim. Acta 27, 31–36. 10.1016/S0003-2670(00)88444-5

[B39] Muyzer G. De Waal E. Uitterlinden A. (1993). Profiling of complex microbial populations by denaturing gradient gel electrophoresis analysis of polymerase chain reaction-amplified genes coding for 16S rRNA. Appl. Environ. Microbiol. 59, 695–700. 10.1128/AEM.59.3.695-700.1993 7683183 PMC202176

[B40] Nautiyal C. S. (1999). An efficient microbiological growth medium for screening phosphate solubilizing microorganisms. FEMS Microbiol. Lett. 170, 265–270. 10.1111/j.1574-6968.1999.tb13383.x 9919677

[B41] Passari A. K. Mishra V. K. Saikia R. Gupta V. K. Singh B. P. (2015). Isolation, abundance and phylogenetic affiliation of endophytic actinomycetes associated with medicinal plants and screening for their *in vitro* antimicrobial biosynthetic potential. Front. Microbiol. 6, 273. 10.3389/fmicb.2015.00273 25904906 PMC4388002

[B42] Payne S. M. (1993). Iron acquisition in microbial pathogenesis. Trends. Microbiol. 1, 66–69. 10.1016/0966-842X(93)90036-Q 8044465

[B43] Pepe O. Ventorino V. Blaiotta G. (2013). Dynamic of functional microbial groups during mesophilic composting of agro-industrial wastes and free-living (N_2_)-fixing bacteria application. Waste Manage. 33, 1616–1625. 10.1016/j.wasman.2013.03.025 23647951

[B44] Qureshi M. A. Ahmad Z. A. Akhtar N. Iqbal A. Mujeeb F. Shakir M. A. (2012). Role of phosphate solubilizing bacteria (PSB) in enhancing P availability and promoting cotton growth. J. Anim. Plant Sci. 22, 204–210.

[B45] Rajput M. S. Naresh Kumar G. Rajkumar S. (2013). Repression of oxalic acid-mediated mineral phosphate solubilization in rhizospheric isolates of *Klebsiella pneumoniae* by succinate. Arch. Microbiol. 195, 81–88. 10.1007/s00203-012-0850-x 23124768

[B46] Reddy P. P. (2013). Recent advances in crop protection (New Delhi: Springer India).

[B47] Romano I. Ventorino V. Pepe O. (2020). Effectiveness of plant beneficial microbes: overview of the methodological approaches for the assessment of root colonization and persistence. Front. Plant Sci. 11, 6. 10.3389/fpls.2020.00006 32076431 PMC7006617

[B48] Rösch C. Mergel A. Bothe H. (2002). Biodiversity of denitrifying and dinitrogen-fixing bacteria in an acid forest soil. Appl. Environ. Microbiol. 68, 3818–3829. 10.1128/AEM.68.8.3818-3829.2002 12147477 PMC124007

[B49] Roy E. D. (2017). Phosphorus recovery and recycling with ecological engineering: a review. Ecol. Eng. 98, 213–227. 10.1016/j.ecoleng.2016.10.076

[B50] Sabaratnam S. Traquair J. A. (2015). Mechanism of antagonism by *Streptomyces griseocarneus* (strain Di944) against fungal pathogens of greenhouse-grown tomato transplants. Can. J. Plant Pathol. 37, 197–211. 10.1080/07060661.2015.1039062

[B51] Sadeghi A. Karimi E. Dahaji P. A. Javid M. G. Dalvand Y. Askari H. (2012). Plant growth promoting activity of an auxin and siderophore producing isolate of *Streptomyces* under saline soil conditions. World J. Microbiol. Biotechnol. 28, 1503–1509. 10.1007/s11274-011-0952-7 22805932

[B52] Saitou N. Nei M. (1987). The neighbor-joining method: a new method for reconstructing phylogenetic trees. Mol. Biol. Evol. 4, 406–425. 10.1093/oxfordjournals.molbev.a040454 3447015

[B53] Schwyn B. Neilands J. B. (1987). Universal chemical assay for the detection and determination of siderophores. Anal. Biochem. 160, 47–56. 10.1016/0003-2697(87)90612-9 2952030

[B54] Sellstedt A. Richau K. H. (2013). Aspects of nitrogen-fixing Actinobacteria, in particular free-living and symbiotic *Frankia*. FEMS Microbiol. Lett. 342, 179–186. 10.1111/1574-6968.12116 23461635

[B55] Shahab S. Ahmed N. Khan N. S. (2009). Indole acetic acid production and enhanced plant growth promotion by indigenous PSBs. Afr. J. Agric. Res. 4, 1312–1316.

[B56] Tiwari M. Ghosh A. Satyapal G. K. Kumar A. (2018). Phosphate solubilization activity of bacterial strains isolated from gangetic plains of north Bihar. Int. J. Biotech. Biochem. 4, 01–08.

[B57] Trabelsi D. Ammar H. B. Mengoni A. Mhamdi R. (2012). Appraisal of the crop-rotation effect of rhizobial inoculation on potato cropping systems in relation to soil bacterial communities. Soil Biol. Biochem. 54, 1–6. 10.1016/j.soilbio.2012.05.013

[B58] Urrutia O. Erro J. Guardado I. Francisco S. S. Mandado M. Baigorri R. (2014). Physico-chemical characterization of humic-metal-phosphate complexes and their potential application to the manufacture of new types of phosphate-based fertilizers. J. Soil Sci. Plant Nutr. 177, 128–136. 10.1002/jpln.201200651

[B59] Vahed H. S. Shahinrokhsar P. Heydarnezhad F. (2012). Performance of phoshate soubilizing bacteria for improving growth and yield of rice (*Oryza sativa* L.) in the presence of phosphorus fertilizer. Int. J. Agric. Crop Sci. 4, 1228–1232.

[B60] Van Oosten M. J. Di Stasio E. Cirillo V. Silletti S. Ventorino V. Pepe O. (2018). Root inoculation with *Azotobacter chroococcum* 76A enhances tomato plants adaptation to salt stress under low N conditions. BMC Plant Biol. 18, 205. 10.1186/s12870-018-1411-5 30236058 PMC6149061

[B61] Ventorino V. Chiurazzi M. Aponte M. Pepe O. Moschetti G. (2007). Genetic diversity of a natural population of *Rhizobium leguminosarum* bv. *viciae* nodulating plants of *Vicia faba* in the Vesuvian area. Curr. Microbiol. 55, 512–517. 10.1007/s00284-007-9024-5 17899266

[B62] Ventorino V. De Marco A. Pepe O. De Santo A. V. Moschetti G. (2012). “Impact of innovative agricultural practices of carbon sequestration on soil microbial community,” in Carbon Sequestration in Agricultural Soils. A Multidisciplinary Approach to Innovative Methods. Ed. Piccolo A. (Berlin, Heidelberg: Springer), 145–177. 10.1007/978-3-642-23385-2_6

[B63] Ventorino V. Parillo R. Testa A. Aliberti A. Pepe O. (2013). Chestnut biomass biodegradation for sustainable agriculture. BioResources 8, 4647–4658. 10.15376/biores.8.3.4647-4658

[B64] Ventorino V. Sannino F. Piccolo A. Cafaro V. Carotenuto R. Pepe O. (2014). *Methylobacterium populi* VP2: plant growth-promoting bacterium isolated from a highly polluted environment for polycyclic aromatic hydrocarbon (PAH) biodegradation. Sci. World J. 2014, 931793. 10.1155/2014/931793 PMC4135167 25152928

[B65] Ventorino V. Ionata E. Birolo L. Montella S. Marcolongo L. de Chiaro A. (2016). Lignocellulose-adapted endo-cellulase producing *Streptomyces* strains for bioconversion of cellulose-based materials. Front. Microbiol. 7, 2061. 10.3389/fmicb.2016.02061 28066379 PMC5177626

[B66] Ventorino V. Robertiello A. Cimini D. Argenzio O. Schiraldi C. Montella S. (2017). Bio-based succinate production from *Arundo donax* hydrolysate with the new natural succinic acid-producing strain *Basfia succiniciproducens* BPP7. Bioenerg. Res. 10, 488–498. 10.1007/s12155-017-9814-y

[B67] Ventorino V. Romano I. Pagliano G. Robertiello A. Pepe O. (2018). Pre-treatment and inoculum affect the microbial community structure and enhance the biogas reactor performance in a pilot-scale biodigestion of municipal solid waste. Waste Manage. 73, 69–77. 10.1016/j.wasman.2017.12.005 29249310

[B68] Viscardi S. Ventorino V. Duran P. Maggio A. De Pascale S. Mora M. L. (2016). Assessment of plant growth promoting activities and abiotic stress tolerance of *Azotobacter chroococcum* strains for a potential use in sustainable agriculture. J. Soil Sci. Plant Nutr. 16, 848–863. 10.4067/s0718-95162016005000060

[B69] Visconti D. Fiorentino N. Cozzolino E. Woo S. L. Fagnano M. Rouphael Y. (2020). Can *Trichoderma*-based biostimulants optimize N use efficiency and stimulate growth of leafy vegetables in greenhouse intensive cropping systems? Agronomy 10, 121. 10.3390/agronomy10010121

[B70] Wei Y. Zhao Y. Shi M. Cao Z. Lu Q. Yang T. (2018). Effect of organic acids production and bacterial community on the possible mechanism of phosphorus solubilization during composting with enriched phosphate-solubilizing bacteria inoculation. Bioresour. Technol. 247, 190–199. 10.1016/j.biortech.2017.09.092 28950126

[B71] Woo S. Pepe O. (2009a). Microbial consortia: Promising probiotics as plant biostimulants for sustainable agriculture, Front. Plant Sci. 9, 1801. 10.3389/fpls.2018.01801 PMC6288764 30564264

[B72] Zaidi A. Khan M. S. Ahemad M. Oves M. Wani P. A. (2009a). “Recent advances in plant growth promotion by phosphate-solubilizing microbes,” in Microbial Strategies for Crop Improvement. Eds. Khan M. Zaidi A. Musarrat J. (Berlin, Heidelberg: Springer), 23–50.

[B73] Zaidi A. Khan M. Ahemad M. Oves M. (2009b). Plant growth promotion by phosphate solubilizing bacteria. Acta Microbiol. Immunol. Hung. 56, 263–284. 10.1556/AMicr.56.2009.3.6 19789141

